# Genetic and genomic analysis modeling of germline *c-MYC *overexpression and cancer susceptibility

**DOI:** 10.1186/1471-2164-9-12

**Published:** 2008-01-11

**Authors:** Xavier Solé, Pilar Hernández, Miguel López de Heredia, Lluís Armengol, Benjamín Rodríguez-Santiago, Laia Gómez, Christopher A Maxwell, Fernando Aguiló, Enric Condom, Jesús Abril, Luis Pérez-Jurado, Xavier Estivill, Virginia Nunes, Gabriel Capellá, Stephen B Gruber, Víctor Moreno, Miguel Angel Pujana

**Affiliations:** 1 Bioinformatics and Biostatistics Unit, and Translational Research Laboratory, Catalan Institute of Oncology, IDIBELL, L'Hospitalet, Barcelona, Spain; 2 Medical and Molecular Genetics Center, IDIBELL, L'Hospitalet, Barcelona, Spain; 3 CIBERER-U730, L'Hospitalet, Barcelona, Spain; 4 Genes and Disease Program, Center for Genomic Regulation, Barcelona, Spain; 5 Genetics Unit, Department of Experimental and Health Sciences, Universitat Pompeu Fabra, Barcelona, Spain; 6 CIBERER-U735, Barcelona, Spain; 7 Department of Urology, Bellvitge Hospital University, IDIBELL, L'Hospitalet, Barcelona, Spain; 8 Department of Pathology, Bellvitge Hospital University, IDIBELL, L'Hospitalet, Barcelona, Spain; 9 Program in Molecular Medicine and Genetics, Vall d'Hebron University Hospital, Barcelona, Spain; 10 Genetic Unit, Department of Physiology II, University of Barcelona, Barcelona, Spain; 11 Departments of Epidemiology, Internal Medicine and Human Genetics, University of Michigan, Ann Arbor, Michigan, USA

## Abstract

**Background:**

Germline genetic variation is associated with the differential expression of many human genes. The phenotypic effects of this type of variation may be important when considering susceptibility to common genetic diseases. Three regions at 8q24 have recently been identified to independently confer risk of prostate cancer. Variation at 8q24 has also recently been associated with risk of breast and colorectal cancer. However, none of the risk variants map at or relatively close to known genes, with *c-MYC *mapping a few hundred kilobases distally.

**Results:**

This study identifies cis-regulators of germline *c-MYC *expression in immortalized lymphocytes of HapMap individuals. Quantitative analysis of *c-MYC *expression in normal prostate tissues suggests an association between overexpression and variants in Region 1 of prostate cancer risk. Somatic *c-MYC *overexpression correlates with prostate cancer progression and more aggressive tumor forms, which was also a pathological variable associated with Region 1. Expression profiling analysis and modeling of transcriptional regulatory networks predicts a functional association between MYC and the prostate tumor suppressor KLF6. Analysis of MYC/Myc-driven cell transformation and tumorigenesis substantiates a model in which MYC overexpression promotes transformation by down-regulating *KLF6*. In this model, a feedback loop through E-cadherin down-regulation causes further transactivation of *c-MYC*.

**Conclusion:**

This study proposes that variation at putative 8q24 cis-regulator(s) of transcription can significantly alter germline *c-MYC *expression levels and, thus, contribute to prostate cancer susceptibility by down-regulating the prostate tumor suppressor *KLF6 *gene.

## Background

Risk of human cancer associated with genetic variation at chromosome 8q24 was first described for prostate cancer in individuals with European ancestry and in African Americans (Risk Region 1) [1,2]. This association was stronger for more aggressive tumor forms [2-4] and for earlier age at diagnosis in African Americans [1,5]. Differences in allele prevalences could account for the higher incidence of prostate cancer in particular populations such as African-Americans [1,2,5]. Subsequently, 8q24 has been associated with risk of prostate cancer by two extra independent regions [6-8] and in risk of breast and colorectal cancer by variation partially overlapping with prostate cancer risk [9-13]. In particular, Haiman *et al.*[12] first noted the existence of common risk variants for breast and colorectal cancer at 8q24. These observations suggest that multiple cancer genes may exist at 8q24 or, alternatively, that risk variants converge on a common biological mechanism [7].

In these studies risk variants did not map to known genes, with few ESTs identified in relatively close proximity. A proposed mechanism includes differences in genomic structure that would make the 8q24 region more prone to subsequent somatic amplification [14]. The *c-MYC *gene is of particular interest in this region because its ectopic expression has been shown to induce prostatic neoplasia [15-17]. Here, we analyze genetic and genomic data to provide evidence of 8q24 cis-regulator(s) of germline *c-MYC *transcription. In addition, genomic data modeling predicts a molecular mechanism linking germline *c-MYC *overexpression and prostate tumorigenesis.

## Results

### Genetic association scan for germline expression differences

**Figure 1:**
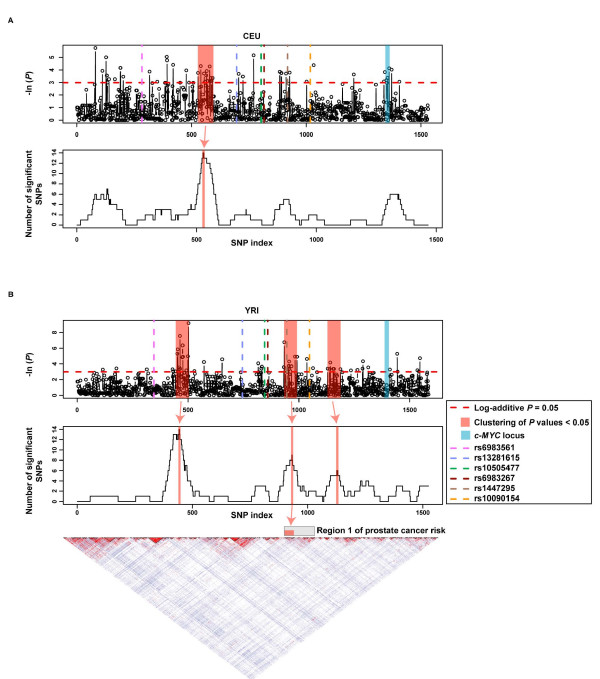
Genetic association scan for germline *c-MYC *differential expression in CEUs and YRIs. (A) Top panel shows results for individual SNPs and bottom panel shows results for significant SNP density in genomic windows of ~42 kb/~66 SNPs in CEUs. The red horizontal dashed line marks the nominal *P *value of 0.05. Variants associated with risk of breast [9], colorectal [10-13] or prostate [1-8, 24] cancer are marked with dashed lines as indicated in the inset. (B) Top panel shows results for individual SNPs and bottom panel shows results for significant SNP density in genomic windows of ~36 kb/~61 SNPs in YRIs. Linkage disequilibrium (*D'/LOD*) plots are shown at the bottom for YRIs. Region 1 of prostate cancer risk is shown.

Scanning associations between genetic variation at 8q24 and *c-MYC *gene expression levels in immortalized lymphocytes of HapMap CEU (Utah residents with ancestry from Northern and WesternEurope) and YRI (Yoruba in Ibadan Nigeria) individuals showed the existence of clusters of SNPs with nominal *P *values < 0.05 (Fig. 1). To assess clustering significance, we examined the proportion of significant SNPs in genomic windows 2- or 4-fold the average size of linkage disequilibrium blocks in CEUs or YRIs, respectively (~42 kb corresponding to ~66 SNPs in CEUs and ~36 kb corresponding to ~61 SNPs in YRIs). Twenty thousand permutations were performed to evaluate the significance of the observed clustering. One genomic region in CEUs and three regions in YRIs were identified with high density of significant SNPs (Fig. 1).

Variation at the *c-MYC *locus was observed with a trend in CEUs, which might suggest the existence of cis-regulators in the gene structural elements (blue bar in Fig. 1A). Two variants in this region (rs4645943 C and rs16902364 A) are associated with germline differential expression of *c-MYC*. The allele frequencies of these SNPs were reported to differ between prostate cancer cases and controls in different populations (i.e. 87.7% (cases) and 77.7% (controls) in Hawaiians; 96.3% (cases) and 95.1% (controls) in CEUs for rs4645943 C) [7]. This observation warrants further genetic analysis of the region with regard to prostate cancer risk.

The scan revealed a possible association between variants in Region 1 of prostate cancer risk and differential germline expression of *c-MYC *(Fig. 1B). Several significant SNPs within this region were identified: the most significant were rs7387447, rs10808558 and rs16902176 (*P *values < 0.01). The rs10808558 A allele showed an association with *c-MYC *overexpression in YRIs (expression difference of 0.23 log_2 _units, 95% confidence interval (CI) 0.06 – 0.41; *P *= 0.007) and this SNP is in low linkage disequilibrium (LD) with the prostate cancer risk variant rs1447295 (r^2 ^= 0.19). Overall, the scan analysis suggests the existence of 8q24 cis-regulators of germline *c-MYC *transcription in lymphocytes, partially overlapping with Region 1 of prostate cancer risk.

### Expression differences in normal prostate tissues

**Figure 2:**
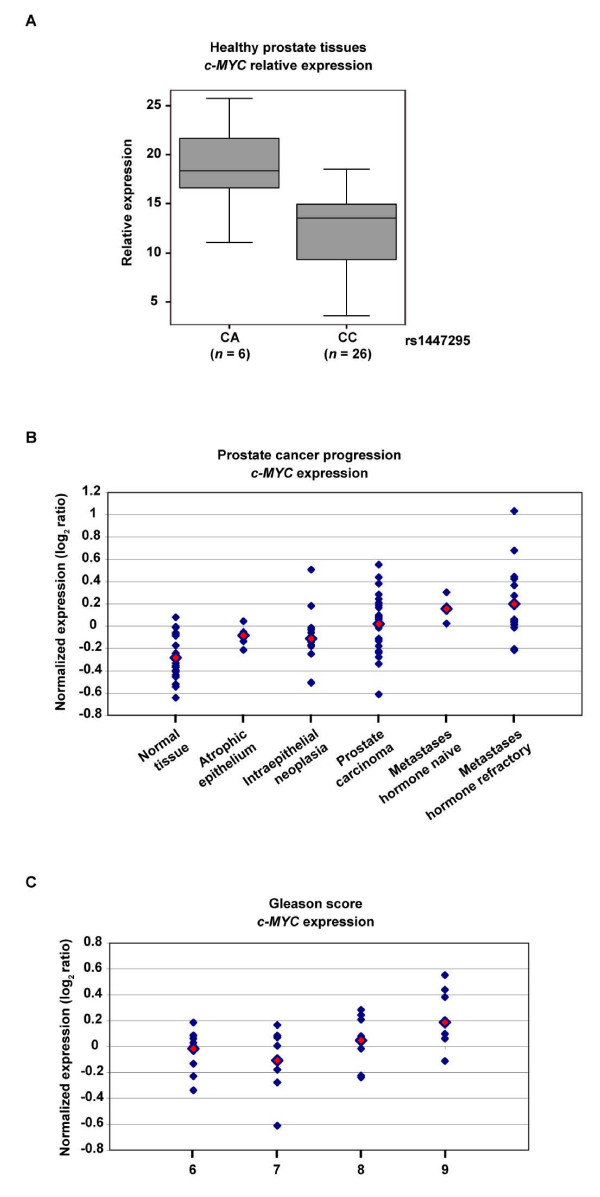
Analysis of *c-MYC *expression in normal and prostate cancer tissues. (A) Relative expression differences of *c-MYC *calculated using three gene references with the following formula: *R *= *F*_*c*-*MYC *_- (*F*_*TBP *_- *F*_*ALAS*1_) where *F*_*gene i*_= *Ct*_*gene i *_- *Ct*_18 *S*_. (B) *c-MYC *expression in prostate cancer progression. Mean expression values are marked by a red solid rhombus. (C) *c-MYC *expression association study with Gleason scores.

Given the possible association of Region 1 variants with germline *c-MYC *overexpression in immortalized lymphocytes of HapMap individuals, we next examined expression differences in normal prostate tissues. For this analysis we used 54 previously characterized normal prostate tissue samples [18,19] and a real-time qRT-PCR protocol developed for prostate samples [20-22]. Genotyping the prostate cancer-associated rs1447295 variant in these samples identified six heterozygotes harboring the risk allele A (CA genotypes). No significant age differences were found between donors harboring the two different genotypes (CA versus CC; no AA homozygotes were identified). Quantitative RT-PCR study using three gene references (*18S*, *ALAS1 *and *TBP*) identified significant *c-MYC *overexpression in samples harboring the risk allele relative to CC homozygotes (n = 26) (Wilcoxon rank sum test *P *= 0.004) (Fig. 2A). In addition, no evidence of allele-specific amplification in tumors arising in CA individuals was observed (not shown). These results suggest the involvement of germline *c-MYC *overexpression in prostate cancer susceptibility.

### Germline copy number variants

As a possible mechanism explaining germline overexpression, we next examined copy number variants (CNVs) at the *c-MYC *locus in CEUs and YRIs, and in 322 unrelated individuals from the Spanish general population using a multiplex ligation-dependent probe amplification (MLPA) assay. This assay identified genomic gains at the *c-MYC *locus at a relatively low frequency in the Spanish general population (< 1%; 2/322) (Additional file 1). However, analysis of rs1447295 genotypes in these individuals did not reveal association with the risk allele and, importantly, none of the CEUs or YRIs showed CNVs with this assay. Therefore, a CNV including *c-MYC *does not seem to be a major contributor to the risk of prostate cancer and germline *c-MYC *overexpression associated with Region 1. Wong *et al.*[23] previously described a CNV including *c-MYC *but only with genomic losses. This observation corroborates the structural complexity of 8q24 and opens the possibility that different genomic configurations are associated with risk alleles in Region 1 or other 8q24 regions.

### Gene expression analysis in prostate tumors

Since Region 1 variants were associated with earlier age at diagnosis and high Gleason scores or aggressive tumor forms [1-8,24], we examined the expression level of 8q24 genes in primary prostate tumors and their association with clinical and pathological variables. For these analyses, we used a publicly available expression data set containing different prostate cellular populations isolated using laser-capture microdissection [25].

Comparison of normal versus neoplastic samples showed differential expression of *c-MYC *(Fig. 2B). Specifically, overexpression appears in the more advanced stages of tumorigenesis such as carcinomas and hormone-refractory metastases (*t*-test *P *< 10^-3^). Tomlins *et al.*[25] previously noted the identification of *c-MYC *in an "overexpressed in progression" signature. The *FAM84B *gene at 8q24 also shows overexpression but mainly at earlier stages (*P *= 0.043 and *P *= 0.002 for intraepithelial neoplasia and carcinomas, respectively), which suggests that *FAM84B *could also be a target of 8q24 somatic amplification. Analysis of Gleason scores showed a trend for *c-MYC *overexpression (ANOVA test *P *= 0.056) (Fig. 2C). Association between *c-MYC *overexpression and high-grade prostate tumors was previously noted by Buttyan *et al.*[26] and Fleming *et al.*[27]. These observations point to a causal relationship between somatic *c-MYC *overexpression and the more aggressive forms of prostate tumors.

### Expression profiles and modeling of transcriptional regulatory networks

**Figure 3:**
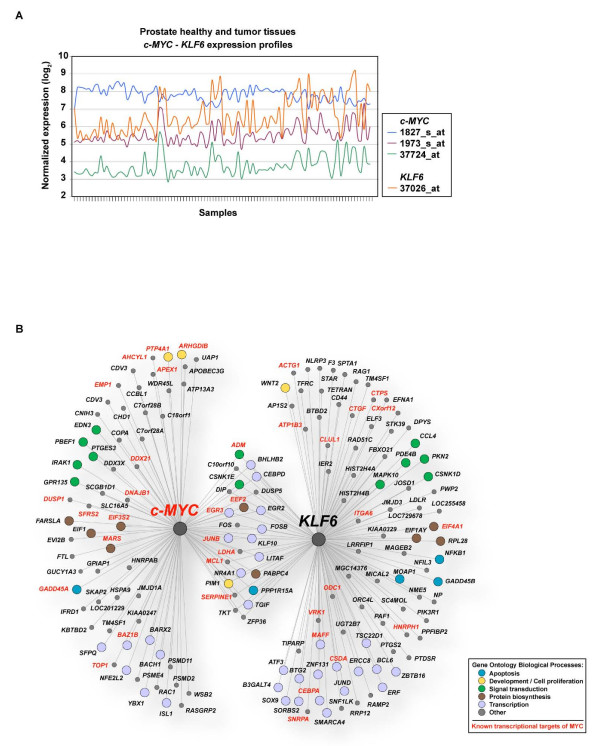
Expression profiling and modeling of transcriptional regulatory networks. (A) Transcriptional profiles of *c-MYC *and *KLF6 *in prostate tissues [30] using U95A Affymetrix probes shown in the inset. (B) Integrated transcriptional regulatory networks of MYC and KLF6. Gene function assignment based on GO term annotations and known MYC transcription targets are shown as indicated in the inset.

Transcriptional targets of MYC include many genes that were identified as conferring risk of prostate cancer and/or being somatically mutated in prostate tumors [28,29]. We sought to identify which of these genes, particularly those conferring risk of prostate cancer, could be functionally associated with *c-MYC *by examining the similarity between expression profiles using a data set containing 50 normal tissues and 52 prostate tumors [30]. This analysis revealed strong correlations between *c-MYC *and the prostate tumor suppressor *Kruppel-like factor 6 (KLF6) *gene (Fig. 3A). Correlations were positive for *c-MYC *microarray probes 1973_s_at and 37724_at (Pearson's correlation coefficient (PCC) = 0.65; *P *< 10^-13^) and negative for 1827_s_at (PCC = -0.71; *P *< 10^-15^). Extensive alternative splicing of the *c-MYC *mRNA could account for this difference [31].

To determine the molecular consequence of the predicted MYC-KLF6 functional association, we generated models of transcriptional regulatory networks in prostate tissues. Using the ARACNe algorithm [32] and the 102 hybridizations of Singh *et al.*[30], we identified 88 and 111 putative transcriptional targets of MYC and KLF6 in this cell type, respectively (Fig. 3B). The intersection of these two sets contains 25 genes, which is a much larger number of genes than randomly expected using simulations of equivalent gene sets (empirical *P *< 0.001). Importantly, 16 of these genes contain MYC binding sites at their promoters based on TRANSFAC (eukaryotes transcription factors database) matrices [33]. In addition, many known MYC targets [29] were also identified: 22 out of 88 (25%) and 23 out of 111 (20%) of the MYC and KLF6 predicted transcriptional targets, respectively (Fig. 3B). Notably, *c-MYC *and *KLF6 *were also directly connected and the *KLF6 *promoter contains three predicted binding sites for MYC (not shown). A 5-gene recurrence predictor of prostate cancer [34] contains *KLF6*, three common ARACNe-based predictions between MYC and KLF6 (*FOS*, *JUNB *and *ZFP36*), and *PPFIA3*, which is functionally related to another predicted target of KLF6 (*PPFIBP2*) (Fig. 3B). These observations further support the role of *KLF6*, *c-MYC *and the ARACNe-based predictions in prostate tumorigenesis.

Comparison of ARACNe-based predictions with the Tomlins *et al.* data set [25] identified 13 of the 88 predicted MYC transcriptional targets differentially expressed between normal prostate tissues and androgen-independent metastases (FDR-adjusted *P *values < 0.05). In addition, 20 of the predicted targets were found to be differentially expressed between normal prostate tissues and adenocarcinomas. Notably, ~46–40% of these genes (6/13 and 8/20) were also predicted to be direct transcriptional targets of KLF6 by the ARACNe algorithm, which endorses the putative functional association between MYC and KLF6.

### Analysis of MYC/Myc-driven cellular transformation and tumorigenesis

**Figure 4:**
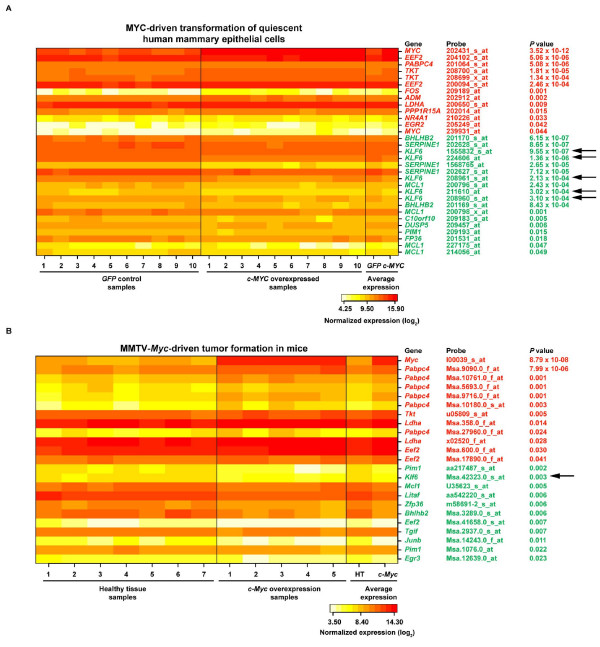
Expression analysis of predicted MYC/KLF6 transcriptional targets in MYC/Myc-driven cell transformation and tumorigenesis. (A) Results of the analysis of quiescent human mammary epithelial cells [36]. (B) Results of the analysis of MMTV-Myc-driven tumors in mice [35]. Genes (red, up-regulated; green, down-regulated), corresponding microarray probes and two-tailed *t*-test *P *values are shown.

To evaluate the functional significance of the predicted shared MYC/KLF6 transcriptional targets, we examined expression data derived from a model of MYC-driven cellular transformation of quiescent human mammary epithelial cells and from MMTV-Myc-driven mammary tumors in mice [35,36]. Of the 25 predicted common targets, 16 (64%) were found to be differentially expressed in cell transformation of quiescent human mammary epithelial cells (Fig. 4A). This proportion is ~2-fold higher than expected by chance taking into account all genes examined in the microarray platform (*χ*^2^-test *P *= 0.004), which substantiates the identification of true MYC targets. Moreover, 11 of the 16 genes contain MYC binding sites at their promoters. Importantly, *KLF6 *was also identified and showed strong down-regulation in this model (*t*-test *P *values < 10^-3^) (Fig. 4A).

Analysis of MMTV-*Myc*-driven mammary tumors in mice showed consistent results with the analysis of quiescent human mammary epithelial cells. Twelve differentially expressed genes were detected, eight of which coincided with the human genes mentioned above (Fig. 4B). Genes that did not overlap between the two studies showed similar trends, for example the human *TGIF *showed a trend for down-regulation (*P *= 0.067) while it was identified as significant in the study of mice tumors (*P *= 0.007). Importantly, this analysis also revealed *Klf6 *down-regulation (*P *= 0.003) (Fig. 4B). Overall, the discovery of *KLF6/Klf6 *down-regulation in two different models of MYC/Myc-driven cell transformation supports the hypothesis that *c-MYC *germline overexpression could act as a risk factor for prostate cancer by converging on a molecular mechanism such as the functional inactivation of the *KLF6 *gene or gene product.

Using the MYC/Myc-driven cell transformation models, we next examined the differential expression of known KLF6 transcriptional targets of relevance to epithelial cancers, E-cadherin (*CDH1 *gene) [37] and p21 (*CDKN1A*) [38]. This analysis revealed strong down-regulation of *CDH1 *in the transformation of quiescent human mammary epithelial cells (*P *values < 10^-5^) and a trend in the model of Myc-driven mice tumorigenesis (*P *= 0.088). No significant differences were appreciable for *CDKN1A *or *Cdkn1a*. These observations suggest that KLF6 down-regulation mediated by germline MYC overexpression could promote epithelial neoplasia by down-regulating E-cadherin.

## Discussion

Combined analysis of genetic and expression data facilitates the identification of transcriptional regulators acting in any part of the genome [39,40]. Examination of different ethnic groups reinforces the identification of these regulators but also reveals differences between populations [41,42]. Due to their functional and structural complexity, transcriptional regulators are largely undercharacterized. However, it is thought that their genetic variability may be relevant when considering susceptibility to common diseases. Specifically, their causal relationship to cancer is almost unknown since most genetic analyses have been focused on coding regions. Insights into differential germline gene expression and tumorigenesis have been gained mainly from mice models, such as the overexpression of the *RAS *family of genes [43], *Mad2 *[44] or *c-MYC *[45,46].

This study analyzed the hypothesis that variation at 8q24 cis-regulator(s) of transcription could significantly alter germline *c-MYC *expression levels and, thus, contribute to cancer susceptibility. Although the genetic scanning analysis performed is susceptible to false positives, the existence of true cis-regulator(s) is suggested by the identification of clusters of significant SNPs. Although larger sample series are required to draw definitive conclusions, the quantitative analysis of geneexpression in normal prostate tissues supports the model of *c-MYC *overexpression associated with Region 1 of prostate cancer risk. Tissue-specific cis-regulator(s) that correlate with additional cancer risk regions at 8q24 may also exist. In a recent study it was noted that tissue specificity is a critical factor in the transcriptional responsiveness of MYC targets [47].

The 8q24 region appears amplified in up to 50% of prostate tumors and *c-MYC *is thought to be the primary target of these amplifications since it is overexpressed in prostate hyperplasia and neoplasia [25]. Ectopic overexpression of *c-MYC/c-Myc *is sufficient to immortalize human prostate epithelial cells [17] and has been shown to generate human-like prostate tumors in mice [16]. In addition, *c-MYC *overexpression in prostate cancer cells enables androgen-independent growth [48]. These observations lead to suggestions of a dual role for *c-MYC *in prostate cancer. At early stages it would promote proliferation while at later stages it would facilitate androgen-independent growth [17]. Our study further proposes that germline *c-MYC *overexpression may promote cellular transformation of the normal epithelium and, by extension, risk of prostate cancer by down-regulating the prostate tumor suppressor *KLF6 *gene. This model is hypothetical and mainly based on the application of the ARACNe algorithm, which achieves a reasonable tradeoff between true- and false-positive rates by eliminating the majority of indirect interactions inferred from gene co-expression [49,50]. Experimental corroboration of the predictions generated in this study is therefore needed, particularly in prostate tissues or cell lines.

The *KLF6 *gene is inactivated in prostate cancer by loss of heterozygosity and/or by somatic mutations identified in tumors, cell lines and xenografts [51]. Recent evidence has extended the role of *KLF6 *inactivation to several other neoplastic processes as esophageal carcinomas [52], glioblastomas [53], head and neck squamous cell carcinomas [54], hepatocellular carcinomas [55], non-small cell lung cancer [56], ovarian carcinomas [57] and particularly, with regard to 8q24 risk variants, to colorectal cancer [58]. A key KLF6 transcriptional target for epithelial neoplasia is E-cadherin (*CDH1 *gene), which is a suppressor of cellular invasion [37]. KLF6 directly transactivates the *CDH1 *promoter resulting in increased levels of its gene product [37]. *CDH1 *is genetically inactivated in many human cancers and shows reduced or absent expression in approximately 50% of prostate tumors [59], playing a critical role in the transition from a noninvasive to an invasive phenotype [60]. Notably, it has recently been proposed that EphB receptors act as tumor suppressors of colorectal cancer, and possibly breast and prostate cancer, through an E-cadherin-mediated mechanism that compartmentalizes tumor cells in the initial stages of tumorigenesis [61]. Loss of E-cadherin can result in β-catenin nuclear localization and, as a result, the up-regulation of LEF/TCF-mediated transcriptional targets such as *c-MYC*[62]. Overall, our study suggests the existence of a transcriptional regulatory circuit that is perturbed in human cancer and which begins with the germline overexpression of *c-MYC*, causing down-regulation of *KLF6 *which then reduces the transactivation of *CDH1*, which in turns feeds *c-MYC *expression through β-catenin and LEF/TCF transcriptional complex activation.

Variants at 8q24 have been associated with risk of prostate, breast and colorectal cancer [1-13,24,63]. Although there are different blocks of linkage disequilibrium that harbor risk variants, cancer clustering might suggests the existence of a common molecular mechanism of susceptibility. Expression analyses in normal prostate, breast and colorectal tissues and examination of association with genotypes are needed to determine the convergence on a common mechanism. Nonetheless, tumor tissue specificity may show dependences on specific, although not fully understood, mechanisms of neoplasia. The ectopic overexpression of *MYC/Myc *in specific cell types of mice promotes breast or prostate tumorigenesis [16,45,64], while widespread expression produces different types of tumors but with preferential appearance of specific epithelial and non-epithelial origins [46]. Overexpression of *c-MYC *also constitutes an early event after loss of the *APC *tumor suppressor gene that initiates colorectal cancer [62,65]. In addition, recent evidence shows that loss of heterozygosity at the *KLF6 *locus contributes to the transition from the compartmentalized carcinoma to the invasive carcinoma, specifically in sporadic colorectal cancer [66,67], which might suggest a link with the mechanism of tumor-cells compartmentalization in the initial stages of tumorigenesis mediated by E-cadherin [61]. Although the predictions generated in this study should be treated with a degree of caution, these observations would agree with the hypothesis of a cancer susceptibility mechanism mediated by *c-MYC *germlineoverexpression.

## Conclusion

This study proposes that variation at putative 8q24 cis-regulator(s) of transcription can significantly alter germline *c-MYC *expression levels and, thus, contribute to prostate cancer susceptibility by down-regulating the prostate tumor suppressor *KLF6 *gene. We propose a transcriptional regulatory model perturbed in human cancer with a feedback loop for *c-MYC*.

## Methods

### Genetic association analysis

We analyzed HapMap genotypes and paired expression data recently made available for immortalized lymphocytes from four ethnic groups and including 210 independent individuals in total (60 Utah residents with ancestry from northern and western Europe; 45 Han Chinese in Beijing; 45 Japanese in Tokyo; and 60 Yoruba in Ibadan Nigeria; Gene Expression Omnibus (GEO) record GSE6536) [42]. Transcriptional differences were scanned between the 128 and 129 Mb of chromosome 8, corresponding to ~1,530 SNPs (NCBI build 35). Scans were performed in R with the SNPassoc package [68]. The log-additive effects of alleles were examined. Association of genotypes with the variable response (gene expression level) was calculated by fitting linear equations and P values obtained by assessing the change in deviance against the null model. Association analysis between genotypes, downloaded from the HapMap data release 21a, and gene expression levels were performed using the web-software SNPStats [69]. The *D'/LOD *plots were generated using the Haploview software [70].

### Microarray gene expression analysis

Using the HapMap lymphocyte expression data [42] and the prostate cancer data of Tomlins *et al.*[25], matrix series were downloaded from GEO references GSE6536 and GSE6099, respectively. Using the Singh *et al.*[30] raw data, background correction, normalization and averaging of expression values were performed with the robust multi-array average (RMA) algorithm. ARACNe Java [49,50] was used to model the gene expression regulatory networks of *c-MYC *and *KLF6*. In this analysis, data processing inequality (DPI) tolerance was set to 0.20 and the mutual information (MI) threshold was 0.05. Normalized data sets of MYC/Myc-driven cellular transformation and tumorigenesis were downloaded from the GEO records GSE3151 and GSE3158 [35,36]. Gene probes were matched using the NetAffx (Affymetrix) tool and differentially expressed probes were identified by calculating two-tailed *t*-test *P *values.

### Genotyping and quantitative RT-PCR analyses

Prostate tissue specimens were collected through the Tumor Bank of the Bellvitge University Hospital and the Catalan Institute of Oncology. Genotyping of rs1447295 was performed by direct sequencing of PCR products of genomic DNA using the following forward and reverse primers, respectively: 5'-GAGTTGCACGCCAGACACTA-3' and 5'-TTTCCCATACCCCATTCTGA-3'. Quantitative RT-PCR analysis of *c-MYC *was performed using a protocol previously developed with the LightCycler™ DNA Master SYBR Green I Kit (Roche Applied Sciences) [20-22] and *c-MYC *primers 5'-CAGCTGCTTAGACGCTGGATT-3' and 5'-GTAGAAATACGGCTGCACCGA-3', and *TBP *primers 5'-GAACCACGGCACTGATTTTC-3' and 5'-CACAGCTCCCCACCATATTC-3'. Relative expression differences were calculated using three gene references (*18S*, *ALAS1 *and *TBP*) with the following formula: *R *= *F*_*c*-*MYC *_- (*F*_*TBP *_- *F*_*ALAS*1_) where *F*_*gene i *_= *Ct*_*gene i *_- *Ct*_18 *S*_.

### Copy number variant analysis

MLPA assays were performed following the conventional protocol with 150 ng of DNA, overnight ligation and 32 cycles of PCR. Probes for *c-MYC *were 5'-GGGTTCCCTAAGGGTTGGAGGAGGAACGAGCTAAAACGGAGCT-3' and 5'P-TTTTTGCCCTGCGTGACCAGATCCTCTAGATTGGATCTTGCTGGCAC-3'.

## Authors' contributions

XS participated in the study design, compiled and analyzed the HapMap data, performed the association analysis and the modeling of transcriptional regulatory networks. PH compiled and analyzed the prostate cancer expression data sets and performed the modeling of transcriptional regulatory networks. FA and EC obtained the tissue specimens. MLH, JA and VN performed the quantitative expression analysis. LA, BRS, LG, LPJ and XE performed the copy number variants analysis. CAM, GC and SBG participated in scientific discussions and helped with the overall interpretation of the data. XS, VM and MAP conceived and designed the study. MAP wrote the original and final versions of the manuscript. All authors read and approved the final version of the manuscript.

## Supplementary Material

Additional file 1 MLPA analysis of several cancer loci including *c-MYC*. Germline genomic gain at *c-MYC *was identified in a sample (bottom) by comparing relative peak intensities. Click here for file
