# Supplementary figures and images for: Genetic and genomic analysis modeling of germline c-MYC overexpression and cancer susceptibility
Source: BMC Genomics. 2008 Jan 11;9:12. doi: 10.1186/1471-2164-9-12 (PMC2244606; doi:10.1186/1471-2164-9-12)

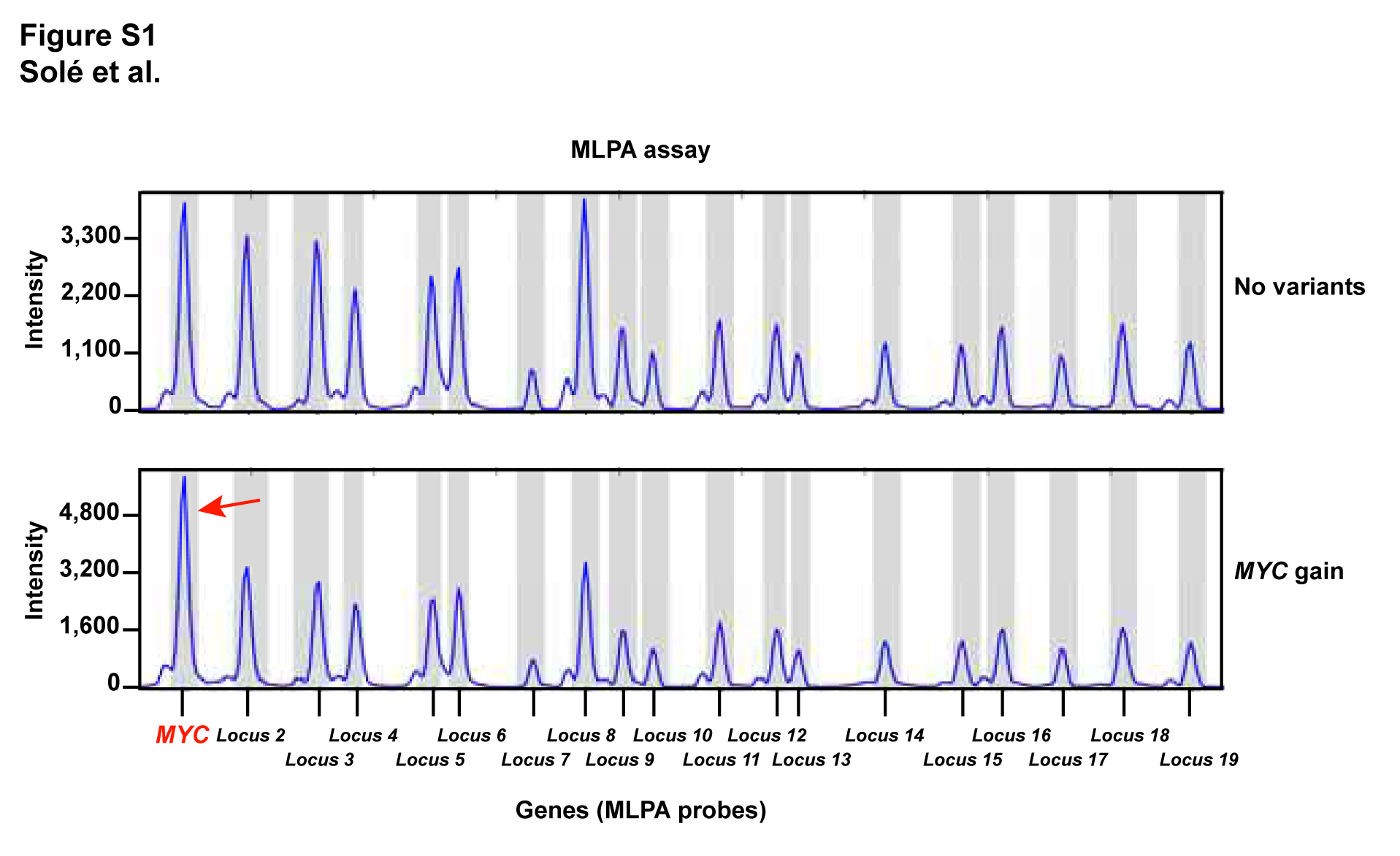

Supplement: Additional file 1 — MLPA analysis of several cancer loci including c-MYC. Germline genomic gain at c-MYC was identified in a sample (bottom) by comparing relative peak intensities. [file 1471-2164-9-12-S1.PNG]
